# Investigating the Gut–Brain Axis in Adolescents with Mood Disorders and Functional Gastrointestinal Symptoms

**DOI:** 10.1192/bjo.2026.11087

**Published:** 2026-06-30

**Authors:** Kwanghun Lee, Sang-Yeol Lee, Bo-Hyun Yoon, Hyung Mo Sung

**Affiliations:** 1 Dr. Lee’s Psychiatric Clinc, Gyeongju, Korea, Republic of; 2 Department of Psychiatry, College of Medicine, Wonkwang University, Iksan, Korea, Republic of; 3 Department of Psychiatry, Naju National Hospital, Naju, Korea, Republic of; 4 Department of Psychiatry, Soonchunhyang University Gumi Hospital, College of Medicine, Soonchunhyang University, Gumi, Korea, Republic of

## Abstract

**Aims:**

This study aimed to estimate the proportion meeting Rome III criteria for functional gastrointestinal disorders (FGIDs) and examine associations with suicidal ideation, depression, anxiety, and perceived stress in child and adolescent psychiatric outpatients, including subgroup analyses by irritable bowel syndrome (IBS), functional constipation (FC), and subtype complexity (single vs. multiple).

**Methods:**

We recruited 137 outpatients aged 12–18 years (2021–2023). FGIDs wereclassified with the Questionnaire on Pediatric Gastrointestinal Symptoms–Rome III (QPGS-Rome III). Beck Scale for Suicide Ideation (BSS), item Center for Epidemiological Studies–Depression Scale for Children (CES-DC), self-report version of the Screen for Child Anxiety Related Emotional Disorders (SCARED), and Perceived Stress Scale (PSS) assessed psychosocial burden. Group differences used standard comparisons (two-sided p<0.05).

**Results:**

Overall, 69.3% met Rome III criteria for ≥1 FGID subtype. Compared with the non-FGID group, the FGID group had higher scores on BSS (p=0.048), CES-DC (p=0.035), SCARED (p<0.001), and PSS (p=0.003). In three-group comparisons (non/single/multiple), CES-DC, SCARED, and PSS differed significantly, with single and multiple FGID groups exceeding non-FGID. In subgroup analyses, IBS-related groups showed higher anxiety and stress than non-FGID, whereas the single FC subtype did not differ significantly.

**Conclusion:**

Among child and adolescent psychiatric outpatients, the proportion meeting the Rome III criteria for FGIDs was high and was significantly associated with psychological burden. These findings support integrating gastrointestinal symptom screening with mental health assessments at intake, with risk-stratified management for IBS-related presentations.

